# Detection and Characterization of Tick-Borne Encephalitis Virus in Baltic Countries and Eastern Poland

**DOI:** 10.1371/journal.pone.0061374

**Published:** 2013-05-01

**Authors:** Olga Katargina, Stanislava Russakova, Julia Geller, Macije Kondrusik, Joanna Zajkowska, Milda Zygutiene, Antra Bormane, Julia Trofimova, Irina Golovljova

**Affiliations:** 1 Department of Virology, National Institute for Health Development, Tallinn, Estonia; 2 Department of Gene Technology, Tallinn University of Technology, Tallinn, Estonia; 3 Department of Infectious Diseases and Neuroinfections, Medical University, Bialystok, Poland; 4 Department of Epidemiological Surveillance, Centre for Communicable Diseases and AIDS, Vilnius, Lithuania; 5 Infectious Diseases Surveillance and Immunisation Unit, Centre for Disease Prevention and Control of Latvia, Riga, Latvia; 6 Molecular Biology of Bacterial Infection Unit, Latvian Centre of Infectious Diseases, East University Hospital, Riga, Latvia; University of Minnesota, United States of America

## Abstract

Ticks were collected from the vegetation in the Baltic countries Estonia, Latvia, Lithuania and eastern Poland and analyzed for the presence of tick-borne encephalitis virus (TBEV) by amplification of the partial E and NS3 genes. In Estonia we found statistically significant differences in the TBEV prevalence between *I. persulcatus* and *I. ricinus* ticks (4.23% and 0.42%, respectively). In Latvia, the difference in TBEV prevalence between the two species was not statistically significant (1.02% for *I. persulcatus* and 1.51% for *I. ricinus*, respectively). In Lithuania and Poland TBEV was detected in 0.24% and 0.11% of *I. ricinus* ticks, respectively. Genetic characterization of the partial E and NS3 sequences demonstrated that the TBEV strains belonged to the European subtype in all countries, as well as to the Siberian subtype in Estonia. We also found that in areas where ranges of two tick species overlap, the TBEV subtypes may be detected not only in their natural vector, but also in sympatric tick species.

## Introduction

Tick-borne encephalitis (TBE) is one of the most important human infections of the central nervous system. The TBE virus causes potentially fatal central nervous system infections, with thousands of cases reported annually throughout central and Eastern Europe and Russia. During 1990–2009 an average of about 8500 human cases were registered per year, of which 2800 per year in Europe [1]. The Baltic countries are considered a TBE endemic area with one of the highest incidence rates in the Europe. The causative agent of the disease is tick-borne encephalitis virus (TBEV), belonging to the genus *Flavivirus* within the family *Flaviviridae*. The TBEV is enveloped virus with a single positive-stranded RNA molecule of approximately 11 kb, containing one open reading frame (ORF), which encodes 10 proteins [2].

Two different types of hosts are needed for the survival of TBEV: ticks that act as both virus vectors and reservoir hosts, and vertebrates which act as a reservoir and a source of blood for feeding ticks and support TBEV transmission by co-feeding of infected and non-infected ticks on the same host [3]. Two tick species, *I. ricinus* (castor bean tick) and *I. persulcatus* (Taiga tick), are the main vectors for TBEV, and belong to the hard tick family (*Ixodidae*). In general, the European TBEV subtype is carried by *Ixodes ricinus* ticks, while the Siberian and the Far Eastern subtypes are carried by *Ixodes persulcatus* ticks [4], [5], [6]. Tick's life cycle varies from two to six years and involves four different stages of development: egg, larva, nymph and adult. All ticks feed only once during each stage and the tick's life span depends on the time intervals between successful feedings as well as climatic conditions [7]. A variety of host animals has been described for *Ixodes* ticks with more than 300 different species of wild and domestic mammals, birds and reptiles. The geographical distribution of *I. ricinus* includes areas of Europe and parts of North Africa [4], [7], *I. persulcatus* has a broad range in Eurasia from the Baltic countries Estonia, Latvia, and Finland in the west to northern Japan in the east [1], [5], [8].

Genetically TBEV is subdivided into the three lineages: the European (TBEV-Eur, Western), the Far-Eastern (TBEV-FE) and the Siberian (TBEV-Sib) subtypes, respectively. Recent studies have shown that the TBEV-Sib and TBEV-FE subtypes are phylogenetically more closely related to each other than to the TBEV-Eur subtype [9]. The distribution of TBEV subtypes corresponds to the ranges of their tick vectors. Thus the TBEV-Eur subtype is widely distributed across Europe comprising strains isolated in Austria, Switzerland, Sweden, Germany, Slovenia, Czech Republic, Hungary, Finland, Estonia, Latvia, Lithuania, Belarus, and the European part of Russia [5], [10]. TBEV-FE and TBEV-Sib strains have been found from Japan and Far East Russia to the Baltic-Nordic countries, Latvia, Estonia and Finland [6], [10], [11], [12].

Previous studies have shown that all three subtypes of TBEV are present in Estonia and Latvia [10], [13], while only the TBEV-Eur subtype has been detected in Lithuania and Poland [11], [14]. The ranges of the two species of TBEV vectors, *I. ricinus* and *I. persulcatus* overlap in the Eastern parts of Estonia and Latvia ([15], Golovljova unpublished data), while only I. ricinus has been found as a TBEV vector in Lithuania and the eastern part of Poland. An additional tick species, *Dermacentor reticulatus*, which is not recognized as an efficient vector for TBEV transmission is also present in the same areas in Lithuania and Poland.

The objectives of the present study were comparison of the TBEV prevalence and distribution in questing ticks collected in Baltic countries and eastern Poland and characterization of local TBEV strains by sequencing of the partial E and NS3 gene regions.

## Materials and Methods

### Tick collection

**Figure 1:**
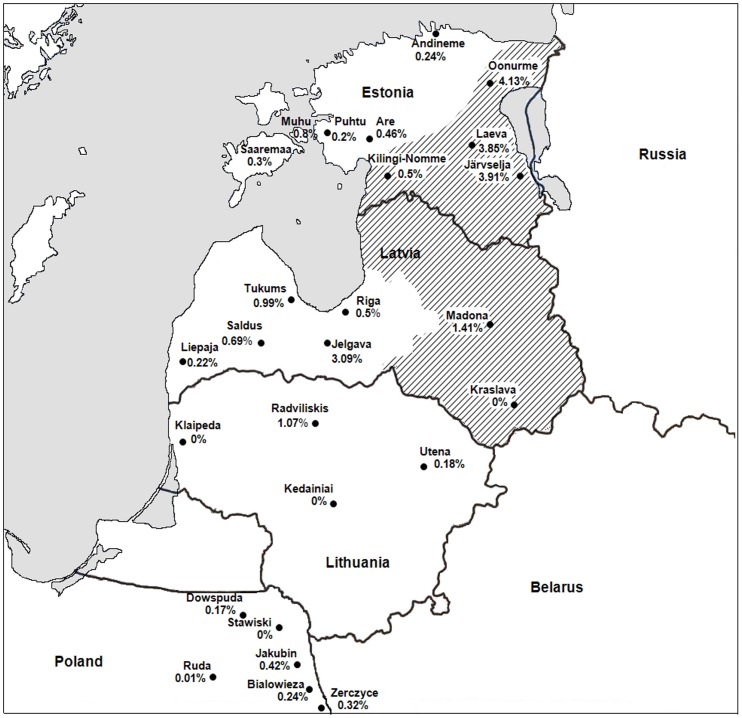
Sites of tick collections, a minimum infection rate (MIR) is shown in percentages. Sympatric area for *I. persulcatus* and *I. ricinus* tick species is dashed according to Karelis [15] and Golovljova unpublished data.

Ticks were collected at 7 sites on mainland Estonia (Laeva 26.446°E, 58.434^o^N; Järvselja 27.305°E, 58.255°N; Andineme 25.458°E, 59.495°N; Oonurme 26.983°E, 59.100°N; Kilingi-Nõmme 24.880°E, 58.164°N; Puhtu 23.551°E, 58.560°N; Are 24.539°E, 58.524°N) on Saaremaa (28.451°E, 58.249°N) and Muhu islands (23.238°E, 58.592°N), at six sites in Eastern Poland (Jakubin 23.180°E, 53.117°N; Bialowieza 23.850°E, 54.700°N; Dowspuda 22.950°E, 54.100°N; Stawiski 21.933°E, 52.417°N; Ruda 22.517°E, 54.033°N; Zerczyce 22.867°E, 52.434°N), at seven sites in Latvia (Kraslava 27.138°E, 56.007°N; Madona 26.486°E, 56.793°N; Jelgava 23.804°E, 56.698°N; Tukums 23.501°E, 57.013°N; Saldus 22.818°E, 56.648°N; Riga 23.790°E, 56.833°N; Liepaja 21.161°E, 56.689°N) and at four in Lithuania (Utena 25.517°E, 55.600°N; Radviliskis 23.617°E, 55.750°N; Klaipeda 21.083°E, 55.750°N; Kedainiai 23.983°E, 55.300°N) (Fig. 1). Sites were chosen in the known TBE-endemic regions and in the transition zone of the two tick species in Estonia and Latvia.

Adults and nymphal ticks were collected from the vegetation monthly from April to November during 2006–2009. At each site a set of four 100 m long line transects were established and flagged by 1-m^2^ flannel cloth. The cloths were examined after every 5 m, all ticks were removed with forceps and maintained alive until later identification.

Species, developmental stage and sex of adult ticks were identified morphologically with a stereo microscope. Collected ticks were investigated individually or pooled into groups of 5 or 10 adults and 5 or 20 nymphs, washed with sterile PBS and stored at −70°C until preparation. Ticks were washed in 70% ethanol and rinsed twice with sterile PBS, homogenized in 400 µl of PBS and stored at −70°C. Two hundred microliters of suspensions were used for RNA extraction.

### RNA extraction

RNA was extracted from 200 µl tick suspension with the guanidinium thiocyanate-phenol-chloroform method, using the TriPure RNA isolation reagent (Roche Diagnostics, Lewes, UK) according to the manufacturer's instructions. RNA was re-suspended in 30 µl of water and stored at −70°C.

### Detection of TBEV

Samples were screened for the presence of specific TBEV RNA by quantitative real-time PCR using primers: F-TBE1 and R-TBE1, and TBE-WT probe as described by Schwaiger and Cassinotti [16] (Table 1). TBEV RNA was amplified in a 25 µl reaction mixture containing of 5 µl of each sample RNA, 12.5 µl of 2X Reaction Mix, 0.5 µl of SuperScript III Platinum One-Step Taq Mix (Invitrogen, USA), 300 nM of forward primer, 900 nM reverse and 250 nM of TBE-WT probe. The cycling conditions comprised 30 min of reverse transcription at 42°C, denaturation for 10 min at 94°C, followed by 45 cycles for 15 sec at 95°C and 1 min at 60°C. The 7500 Fast Real Time PCR system (Applied Biosystems) was used for PCR reactions and fluorescent detections.

**Table 1:** Primers and probe used for the detection of TBEV in Real Time PCR and nested PCR.

Primers/probe	Primer sequence (5′→3′)	References
F-TBE 1	GGG CGG TTC TTG TTC TCC	[16]
R-TBE 1	ACA CAT CAC CTC CTT GTC AGA CT	[16]
TBE-WT	FAM-TGA GCC ACC ATC ACC CAG ACA CA-TAMRA	[16]
283F1	GAG A(T/C)C AGA GTG A(T/C)C GAG GCT GG	[43]
827R1	AGG TGG TAC TTG GTT CC(A/C) TCA AGT	[43]
349F2	GTC AAG GCG (T/G)CT TGT GAG GCA A	[43]
814R2	TTC C(C/A)T CAA TGT G(T/C)G CCA CAG G	[43]
NS3 F1	G(A/G)A A(T/C)G G(C/A)C T(A/G)A A(A/G)A C(T/C)A ATG A	This study
NS3 R1	TGA GCT C(A/G)A C(T/C)(T/C) (T/G)CC C(A/G)T CAA	This study
NS3 F2	TA(T/C) GTC AGC AGC ATT GCT CA	This study
NS3 R2	TTG ATG TTT GT(T/C) C(T/G)G (T/C)TC CAT CTA T	This study
16Sa	CGCCTGTTTATCAAAAACAT	[17]
16Sb	CTCCGGTTTGAACTCAGATC	[17]

Samples positives by real-time PCR were used for one step RT-PCR and for nested PCR for future sequencing of the partial E protein gene with outer primers: 283F1 and 827R1 and with inner primers: 349F2 and 814R2, and for the NS3 gene with outer primers NS3 F1 and NS3 R1 and inner primers NS3 F2 and NS3 R2 (Table 1). RT-PCR amplification was carried out in a total reaction volume of 25 µl, which contained the following mix of reagents: 2x Reaction mix (Invitrogen, Carlsbad USA), 0.2 µM of forward and reverse primers, 1 µl of SuperScript® III RT/Platinum® *Taq* Mix (Invitrogen, Carlsbad USA), and 5 µl of target RNA. The cycling conditions comprised cDNA synthesis 30 min at 50°C, denaturation for 2 min at 94°C, followed by 40 cycles for 20 sec at 94°C, 1 min at 60°C for E gene and 55°C for NS3 gene, and 1 min at 68°C, followed by an extension 68°C for 5 min.

The nested PCR amplifications were performed in a total volume of 50 µl as follows: GeneAmp 10xPCR buffer II, MgCl_2_ (1.5 mM for E gene, 5 mM for NS3 gene) 600 µM for E gene amplification, 800 µM for NS3 gene of dNTPs, 0.5 mM of forward and reverse primers, AmpliTaq DNA polymerase (2.5U) (Applied Biosystems, Roche, Branchburg, NJ), and 5 µl of target DNA from the first PCR reaction. The cycling conditions were an initial denaturation for 2 min at 94°C, followed by 30 cycles for 1 min at 94°C, 1 min at 65°C for the E gene and 55°C for the NS3 gene, and 1 min at 72°C, followed by an extension at 72°C for 10 min.

Products of the nested PCR were purified with QIAquick PCR purification kit (QIAGEN, Hilden, Germany) according to the manufacturer's instructions. The BigDye Terminator v3.1 Cycle sequencing kit (Applied Biosystems, Forest City, CA, USA) was used for the DNA sequencing reaction according to the manufacturer's recommendations, followed by sequencing on the ABI PRISM 3100 Genetic Analyser (Applied Biosystems). The obtained sequences were edited using the BioEdit program (www.mbio.ncsu.edu/BioEdit/bioedit.html).

### Confirmation of tick species

The morphological species detection was further confirmed by *Ixodes* mitochondrial 16S RNA PCR and sequencing [17].

PCR amplification was carried out in a total reaction volume of 50 µl, which contained GeneAmp 10xPCR buffer II, MgCl_2_ (1.5 mM) 600 µM of dNTPs, 200 nM of forward and reverse primers, AmpliTaq DNA polymerase (5U) (Applied Biosystems, Roche, Branchburg, NJ), and 5 µl of target DNA. The cycling conditions consisted of 2 cycles of touchdown program, consisting of 1 min of denaturation 94°C, 1 min at annealing temperature decreased from 49°C to 47°C in each cycle, and an extension step of 2 min at 72°C, followed by 40 cycles of a denaturing step of 30 sec at 94°C, an annealing step of 1 min at 45°C, and an extension step of 2 min at 72°C, followed by an extension at 72°C for 10 min.

### Phylogenetic analysis

Sequences were retrieved from the GenBank database and aligned manually using the BioEdit program [18]. The Maximum Likelihood model was used for phylogenetic tree reconstruction using the Tree Puzzle 5.2 version package [19] and 25 000 puzzling steps with Quartet Puzzling (QP) support values >70% were applied using the Hasegawa-Kishino-Yano (HKY) model of substitutions [20]. The transition/transversion ratio and nucleotide frequencies were estimated from data set. GenBank accession numbers of the sequences used in the phylogenetic analysis are given in Table S1.

### Statistical analysis

Collected ticks were analyzed individually as well as in pools of different sizes and the TBEV prevalence in ticks was calculated as a minimum infection rate (MIR) with the assumption that only one tick in each pool was positive.

MIR was analyzed according to sampling site and tick species, and the 95% binomial confidence interval with continuity correction was calculated [21]. Differences between groups were calculated by Fisher's exact test 2×2 contingency table [22].

## Results

### Tick collection and TBEV prevalence in questing ticks

#### Estonia

In this study a total of 3287 ticks from different parts of Estonia were analyzed. Of these, 2341 were identified as *I. ricinus* (1295 adult ticks and 1046 nymphs) and 946 as *I. persulcatus* (588 adult ticks and 358 nymphs). *I. ricinus* ticks were collected throughout the territory of Estonia, whereas *I. persulcatus* ticks were found only in the eastern and south-eastern parts (Laeva, Järvselja, Oonurme and Kilingi-Nõmme). Eastern Estonia is a sympatric distribution area for both tick species with of *I. persulcatus* predominating at 78%, 87% and 93% in Laeva, Oonurme and Järvselja, respectively. Although the site Kilingi-Nõmme is also situated in a sympatric area, the proportion of collected *I. persulcatus* ticks was only 1.5%.

All collected ticks were tested for the presence of TBEV by real-time PCR and 51 Estonian ticks out of 3287 were found positive with an overall MIR of 1.55% (Table 2). Presence of TBEV was found at all analyzed sites and ranged from 0.2% in Puhtu to 4.13% in Oonurme (Table 2). *I. persulcatus* ticks demonstrated a statistically significant difference for TBEV prevalence as compared to *I. ricinus*, with 4.23% (40/946) vs. 0.46% (11/2341) (P<0.0001), respectively. The TBEV prevalence in adult and nymphal ticks within each species did not, however, reveal significant differences (0.46% and 0.48% for *I. ricinus* and 4.60% and 3.63% for *I. persulcatus*, respectively).

**Table 2:** Detection of TBEV in ticks in the Baltic countries.

	*I. ricinus*						*I. persulcatus*							
Location	No. adults infected/tested (MIR%)*	95% CI‡	No. nymphs infected/tested (MIR%/)	95% CI	Total no. ticks infected/tested (MIR%)	95% CI	No. adults infected/tested (MIR%)	95% CI	No. nymphs infected/tested (MIR%)	95% CI	Total no. ticks infected/tested (MIR %)	95% CI	Total no. ticks infected/tested (MIR%)	95% CI
**Estonia**														
Laeva	0/47	-	2/43 (4.65)	0.81–17.05	2/90 (2.22)	0.39–8.55	5/166 (3.01)	1.11–7.26	9/160 (5.63)	2.77–10.74	14/326 (4.30)	2.46–7.26	16/416(3.85)	2.29–6.31
Järvselja	-	-	1/32 (3.13)	0.16–18.01	1/32 (3.13)	0.16–18.01	12/225 (5.33)	2.91–9.36	4/178 (2.24)	0.72–6.03	16/403 (3.97)	2.36–6.5	17/435(3.91)	2.37–6.31
Oonurme	0/10	-	0/21	-	0/31	-	10/191 (5.24)	2.69–9.7	0/20	-	10/211 (4.74)	2.43–8.8	10/242(4.13)	2.11–7.69
K-Nõmme	1/194 (0.52)	0.03–3.2	1/200 (0.50)	0.03–3.18	2/394 (0.51)	0.09–2.03	0/6	-	-		-		2/400 (0.50)	0.09–2
Puhtu	0/200		1/305 (0.33)	0.02–2.1	1/505 (0.20)	0.01–1.28	-	-	-		-		1/505 (0.20)	0.01–1.28
Are	2/202 (0.99)	0.17–3.91	0/230	-	2/432 (0.46)	0.08–1.84	-	-	-		-		2/432 (0.46)	0.08–1.84
Andineme	1/204 (0.49)	0.03–3.12	0/215	-	1/419 (0.24)	0.01–1.54	-	-	-		-		1/419 (0.24)	0.01–1.54
Saaremaa island	1/314 (0.32)	0.02–2.04	-	-	1/314 (0.32)	0.02–2.04	-	-	-		-		1/314 (0.32)	0.02–2.04
Muhu island	1/124 (0.81)	0.04–5.08	-	-	1/124 (0.81)	0.04–5.08	-	-	-		-		1/124 (0.81)	0.04–5.08
**Total**	**6/1295(0.46)**	**0.19**–**1.06**	**5/1046 (0.48)**	**0.18**–**1.18**	**11/2341 (0.46) ^a^**	**0.25**–**0.87**	**27/588 (4.60)**	**3.1**–**6.7**	**13/358 (3.63)**	**2.03**–**6.28**	**40/946 (4.23) ^b^**	**3.08**–**5.77**	**51/3287 (1.55)**	**1.17**–**2.05**
**Latvia**														
Kraslava	0/206	-	0/221	-	0/427	-	0/4	-	0/5	-	0/9	-	0/436	-
Madona	0/30	-	1/117 (0.84)	0.04–5.36	1/147 (0.68)	0.04–4.3	4/166 (2.41)	0.77–6.45	1/112 (0.89)	0.05–5.59	5/278 (1.8)	0.6–4.39	6/425 (1.41)	0.57–3.2%
Jelgava	20/361 (5.54)	3.5–8.57	1/318 (0.31)	0.02–2.01	21/679(3.09)	1.97–4.77	-		-		-		21/679(3.09)	1.97–4.77
Tukums	4/304 (1.31)	0.4–3.57	6/705 (0.85)	0.3–1.94	10/1009 (0.99)	0.5–1.88	-		-		-		10/1009 (0.99)	0.5–1.88
Saldus	2/154 (1.30)	0.2–5.1	0/137	-	2/291 (0.69)	0.12–2.74	-		-		-		2/291 (0.69)	0.12–2.74
Riga	4/301 (1.33)	0.4–3.6	0/501	-	4/802 (0.50)	0.16–1.37	-		-		-		4/802 (0.50)	0.16–1.37
Liepaja	0/100	-	1/357 (0.28)	0.01–1.8	1/457 (0.22)	0.01–1.41	-		-		-		1/457 (0.22)	0.01–1.41
**Total**	**30/1456 (2.06)^ a^**	**1.42**–**2.97**	**9/2356(0.38)^b^**	**0.19**–**0.76**	**39/3812 (1.02)**	**0.74**–**1.41**	**4/170 (2.35)**	**0.75**–**6.3**	**1/117 (0.85)**	**0.04**–**5.36**	**5/287 (1.74)**	**0.64**–**4.25%**	**44/4099 (1.07)**	**0.79**–**1.45**
**Lithuania**														
Utena	0/280	-	1/275 (0.36)	0.2–2.32	1/555 (0.18)	0.01–1.16	-		-		-		1/555 (0.18)	0.01–1.16
Radviliskis	3/190 (1.58)	0.41–4.92	2/275 (0.73)	0.13–2.89	5/465 (1.07)	0.4–2.65	-		-		-		5/465 (1.07)	0.4–2.65
Klaipeda	0/280	-	0/275	-	0/555	-	-		-		-		0/555	-
Kedainiai	0/170	-	0/245	-	0/415	-	-		-		-		0/415	-
**Total**	**3/920(0.32)**	**0.09**–**1.04**	**3/1070 (0.28)**	**0.07**–**0.89**	**6/1990 (0.30)**	**0.12**–**0.69**	**-**		**-**		**-**		**6/1990(0.30)**	**0.12**–**0.69**
**Poland**														
Jakubin	0/560	-	5/640 (0.78)	0.29–1.92	5/1200 (0.42)	0.16–1.03	-		-		-		5/1200(0.42)	0.16–1.03
Bialowieza	1/560 (0.18)	0.01–1.15	2/645 (0.31)	0.05–1.24	3/1205 (0.24)	0.06–0.79	-		-		-		3/1205(0.24)	0.06–0.79
Dowspuda	2/625 (0.32)	0.06–1.28	0/540	-	2/1165 (0.17)	0.03–0.69	-		-		-		2/1165(0.17)	0.03–0.69
Stawiski	0/650	-	0/550	-	0/1200	-	-		-		-		0/1200	
Ruda	0/640	-	1/640 (0.16)	0.01–1.01	1/1280 (0.01)	0–0.51	-		-		-		1/1280(0.01)	0–0.51
Zerczyce	3/570 (0.53)	0.14–1.67	1/650 (0.15)	0.01–0.99	4/1220 (0.32)	0.11–0.9	-		-		-		4/1220(0.32)	0.11–0.9
**Total**	**6/3605(0.17)**	**0.07**–**0.39**	**9/3665 (0.25)**	**0.12**–**0.49**	**15/7270 (0.21)**	**0.12**–**0.35**	**-**		**-**		**-**		**15/7270(0.21)**	**0.12**–**0.35**

* MIR – minimum infection rate %.

‡ 95% CI –95% binominal confidence interval including continuity correction by Wilson [21].

- not detected.

a, b Statistically significant differences (P<0.0001).

In areas sympatric for the two tick species we found that *I.ricinus* ticks demonstrated a statistically higher TBEV prevalence rate (2.22% in Laeva, and 3.13% in Järvselja) than Western Estonia, where the prevalence rate ranged from 0.20 to 0.99% (Table 2).

#### Latvia

Of 4099 analyzed ticks collected in Latvia, 3812 were identified as *I. ricinus* (1456 adult ticks and 2356 nymphs) and 287 as *I. perculcatus* (170 adult ticks and 117 nymphs). *I. persulcatus* ticks were collected only in two out of seven sites (Kraslava and Madona). The proportion of *I. persulcatus* in Kraslava was low, as only single ticks (9 out of 436) were collected. In the second site (Madona), 65.4% (278 out of 425) of ticks were identified as *I. persulcatus*.

Overall, the MIR of TBEV in the analyzed ticks from Latvia was similar to that in Estonia –1.07% (44/4099). TBEV was detected at six sites out of seven and prevalence ranged from 0.22% in Liepaja to 3.09% in Jelgava (Table 2). However, in contrast to the Estonian prevalence rates, Latvian *I. persulcatus* ticks did not show any statistically significant differences compare to *I. ricinus*, with rates of 1.74% (5/287) and 1.02% (39/3812), respectively. The numbers of collected and analyzed *I. persulcatus* ticks were, however, more than 13 times lower than those for *I. ricinus*. We found statistically significant differences (P<0.0001) of TBEV prevalence between adults and nymphs of *I. ricinus*, at 2.06% (30/1456) and 0.38% (9/2356), respectively, while no such differences could be demonstrated for *I. persulcatus* ticks (Table 2).

#### Lithuania

A total of 1990 *I. ricinus* ticks from Lithuania were collected and analyzed, among them 920 adults and 1070 nymphs. Presence of TBEV was detected only at two sites out of four with an overall MIR 0.30%, ranging from 0.18% (Utenos) to 1.07% (Radviliskio). The TBEV infection rate for adults and nymphs did not show any statistical differences (0.32% and 0.28%, respectively).

#### Poland

In Poland, 7270 *I. ricinus* ticks (3605 adults and 3665 nymphs) were collected and analyzed. In addition to *I. ricinus,* 600 adults of *D. reticulatus* ticks were analyzed for the presence of TBEV. Positive ticks were detected at five out six sites, with an overall MIR 0.21% in *I. ricinus* ticks and ranging from 0.01% (Ruda) to 0.42% (Jakubin) (Table 2). Infection rates in adults and nymphal stages were found to be similar (0.17% and 0.25%, respectively).

TBEV RNA was found in two out of 600 *D. reticulatus* adult ticks with an overall MIR of 0.33%.

### Genetic analyses of TBEV sequences

In the present study, 31 samples from ticks collected in Estonia, five samples from Latvia, 2 from Lithuania and eight from Poland were amplified and sequenced for the partial E and/or NS3 genes. A total of 36 samples were analyzed for both genetic regions, and ten samples were sequenced either in the partial E glycoprotein or NS3 genes. TBEV sequences were amplified from *I. persulcatus* and *I. ricinus* from Estonia and Latvia, from *I. ricinus* from Lithuania and from *I. ricinus* and *D. reticulatus* from Poland.

In the Estonian samples, 28 sequences were identified as TBEV-Sib subtype, 25 of which were detected in *I. persulcatus* and, unexpectedly, three in *I. ricinus* (Est1039, Est3512-1, and Est3974), while TBEV-Eu subtype was confirmed in three samples amplified from *I. ricinus*.

Two sequences belonging to TBEV-Sib subtype from *I. ricinus* (Est1039, Est3512-1) were amplified from ticks collected in areas where *I. persulcatus* ticks are prevalent, while one sequence (Est3974-1) originated from the Andineme site, where only *I. ricinus* was collected, and which is located 300 km apart from the *I. persulcatus* range.

The Estonian TBEV-Sib subtype sequences amplified in the present study were either identical to each other or shared a high degree of similarity (up to 99.2%) for the partial E glycoprotein and NS3 genes.

**Figure 2:**
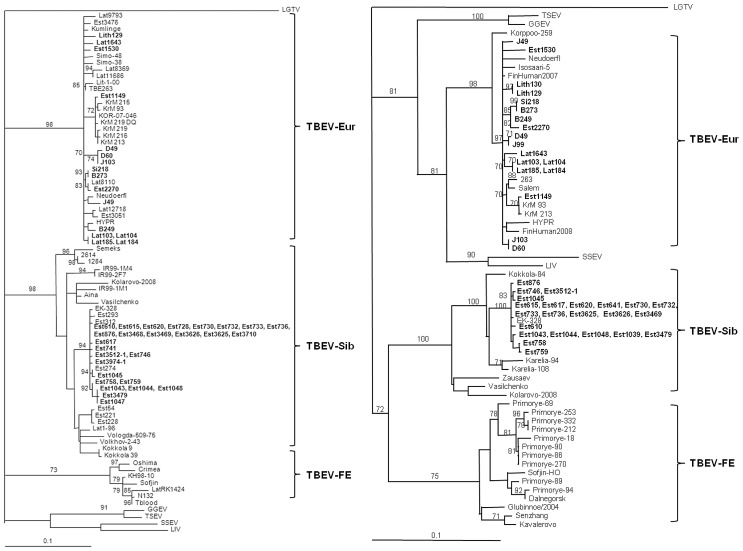
Phylogenetic tree (Maximum Likelihood) based on the partial E short gene (367 bp) sequences (A) and on the NS3 protein encoding sequences (631 bp) (B) of TBEV. Sequences detected in the present study are shown in bold. Only support values exceeding 70% are shown. GenBank accession numbers of the sequences are given in Table S1.

On phylogenetic trees based on the partial NS3 and E genes, the Estonian samples of TBEV-Sib subtype clustered together with strains isolated from *I. persulcatus* in Finland and Karelia (Fig. 2) and demonstrated 96.1–99.6% and 95.5–99.7% similarity rates for NS3 and E genes, respectively. The partial E glycoprotein genes of three other strains belonging to the TBEV-Sib subtype, and isolated from *I. ricinus* have been deposited in GenBank: strain Volkhov-2-43 (FJ214148) isolated in 1943 in Volkhov (approx. 500 km distance from the place of detection of Estonian strains), strain Vologda-509-75 (FJ214142) isolated in the European part of Russia in 1975 (approx. 800 km from Estonia) and strain Semeks (AF224665) isolated in Zhitomir region in Ukraine (approx. 900 km from Estonia). The comparison of the partial E gene nucleotide similarity of Volkhov-2-43 and Vologda-509-75 strains with the Estonian TBEV-Sib sequences also amplified from *I. ricinus* (Est3512-1, Est3974-1) showed the same level of nucleotide substitution as detected within the Baltic sublineage of TBEV-Sib subtype. Strain Semeks was less related to strains isolated in Estonia and the European part of Russia, and clustered together with strains isolated in Siberia. The nucleotide similarities between the Baltic and Siberian sublineages of the TBEV-Sib subtype, were 92.2–95.8% and 92.6–95.0% for partial NS3 and E genes, respectively.

Three sequences for the partial E and NS3 genes (from strains Est1149, Est1530, and Est2270) belonging to the TBEV-Eur subtype were amplified from *I. ricinus* ticks: two from ticks collected in *I. ricinus* allopatric area (Est1530, Est2270) and one (Est1149) collected in an area of mixed range of *Ixodes* species where strains of the TBEV-Sib subtype were predominant. Surprisingly, the partial E glycoprotein and NS3 gene sequences of this strain were more closely related to strains isolated in Korea 98.9–99.7% and 99.6–99.8%, respectively, than with strains circulating in Estonia or other parts of Europe with nucleotide sequence similarities of 96.7–99.1% and 96.5–98.4%, respectively. On the phylogenetic tree based on the partial E glycoprotein gene sequences, the Korean and Estonian strains clustered together and formed a lineage with a high QP support value (Fig. 2).

The two other Estonian *I. ricinus* derived samples (Est1530, Est2270) were randomly distributed within the TBEV-Eur subtype on phylogenetic trees based on the partial E glycoprotein and NS3 genes and demonstrated 97.0–99.1% and 96.8–99.3%, similarity, respectively, with other European strains.

At the Latvian site Madona, where *I. ricinus* and *I. persulcatus* ranges overlap, TBEV was detected in *I. persulcatus* (Lat1643) and surprisingly identified as TBEV-Eur subtype by sequencing of the partial E glycoprotein and NS3 genes. At site Jelgava, where only *I. ricinus* is found, four identical sequences (Lat103, Lat104, Lat184, Lat185) belonging to the TBEV-Eur subtype were detected by amplification of both genetic regions.

In neighboring Lithuania, only *I. ricinus* ticks are present, and two identical sequences (Lith129, Lith130) of the partial E glycoprotein and NS3 genes belonging to the TBEV-Eur subtype were amplified.

At the Polish site Jakubin, TBEV sequences of the partial E glycoprotein and NS3 genes belonging to the TBEV-Eur subtype were detected in *I. ricinus* (J49, J99, J103) as well as in *D. reticulatus* (D49, D60) ticks. At two other sites, Zerczyce (Si218) and Bialowieza (B249, B273) sequences of TBEV-Eu subtype were amplified only from *I. ricinus.*

On the phylogenetic trees based on the partial NS3 and E glycoprotein genes, sequences of TBEV-Eur subtype obtained from Latvian, Lithuanian and Polish ticks did not show geographical clustering within the TBEV-Eur subtype, but demonstrated a high level of nucleotide similarity (97.2–99.8% and 96.0–99.7%, for the partial E glycoprotein and NS3 genes, respectively).

### PCR detection of tick species by sequencing of mitochondrial 16S rRNA gene

In areas where ranges of *I. ricinus* and *I. persulcatus* overlap, morphological identification of *Ixodes* species was confirmed by amplification and subsequent sequencing of the partial mitochondrial 16S rRNA gene (339 bp). The results confirmed that all tick species were correctly identified by the morphological method.

## Discussion

In the present study, the prevalence of TBEV RNA in questing ticks collected from the vegetation in three Baltic countries and Poland was estimated. The reported overall infection rate of TBEV in questing ticks in Estonia (1.55%) and Latvia (1.07%) were statistically higher (P<0.0001) than in *I. ricinus* in Lithuania (0.30%) and Poland (0.21%). In the eastern areas of Estonia and Latvia, the range of *I. ricinus* overlaps with *I. persulcatus,* and the present study showed that *I. persulcatus* ticks more frequently infected by TBEV (4.23% in Estonia and 1.74% in Latvia) than *I. ricinus* ticks (0.46% in Estonia and 1.02% in Latvia). The difference was less pronounced in Latvia as the number of collected *I. persulcatus* ticks was relatively high in only one Latvian site (Madona). Moreover, *I. ricinus* had higher rates of TBEV prevalence in areas sympatric with *I. persulcatus* than in areas where only *I. ricinus* is distributed. These differences in TBEV prevalence rates may reflect more favorable conditions for TBEV circulation in Eastern Estonia, where ranges of the two tick species overlap. In other studies, the TBEV prevalence reported in *I. per*s*ulcatus* ticks varied from 1.0 to 4% in Western Siberia [23] to 6% in Finland [12]. In Latvia, the previously reported TBEV infection rate for *I. per*s*ulcatus* was 5% [24].

In the present study, the infection rate of *I. ricinus* ticks varied from 0.21% in Poland to 1.17% in Latvia. In other European countries the overall reported prevalence rates of TBEV in *I. ricinus* were 0.5%–2.0% in Bavaria [25], 0.2%–1% in Finland [26], and 0.47% in Slovenia [27]. Infection rates of 0.1%–1.7% and similar to the ones in the present study were previously reported from Lithuania [28], while a higher prevalence of 2.4%–3.7% and 1.6% were reported from Latvia and Poland, respectively [14], [24]. The differences in TBEV prevalence rates in different countries may, however, be explained by different methods of virus detection in ticks as well as fluctuations in TBEV prevalence during collection seasons and years.

In the present study we found that strains of TBEV-Sib and TBEV-Eur subtypes may be exchanged between sympatric tick species in the same area. In Estonia we found strains of TBEV-Sib subtype not only in *I. persulcatus,* as demonstrated in previous studies [13], [29], but also in *I. ricinus*. Although *I. ricinus* is considered to be the principal vector for TBEV-Eur subtype in Europe, detection of TBEV-Sib in *I. ricinus* has been reported from the European part of Russia [30]. The retrospective study of strain Volkhov-2-43, isolated from *I. ricinus* in 1943 in Leningrad oblast showed that it belonged to the TBEV – Sib subtype, and to date it is the oldest isolated TBEV-Sib strain [30], [31]. Although this strain was isolated almost 70 years ago, it showed the same level of nucleotide similarity of the partial E glycoprotein gene with other strains of the Baltic lineage within TBEV-Sib subtype isolated from *I. persulcatus* as well as from *I. ricinus*. Estonian sequences of TBEV-Sib subtype amplified from *I. ricinus* also were closely related to or identical on the nucleotide and amino acid levels to other Estonia TBEV-Sib strains isolated from *I. persulcatus*.

A recent study of hemagglutinating deficient TBEV-Sib strains of from the European part of Russia revealed three unique amino acid substitutions in the E glycoprotein, which increased the net charge and hydrophobicity of the virion surface [32]. Increasing hydrophobicity was proposed to be an adaptation of *I. persulcatus* TBEV strains to a new vector i.e. *I. ricinus* [32]. However, the partial E glycoprotein amino acid alignment of Estonian TBEV-Sib sequences amplified from *I. ricinus* was identical to sequences from *I. persulcatus* in the same area and did not show any amino acid substitutions. On the phylogenetic trees based on the partial E glycoprotein and NS3 genes, TBEV-Sib strains from *I. ricinus* also did not form their own lineages, which could be regarded as evidence of adaptation to the new vector, but rather clustered randomly within the Baltic sublineage.

We suggest that strains of TBEV-Sib subtype may infect *I. ricinus* larvae during co-feeding with *I. persulcatus* nymphs on mammals, and that detection of TBEV-Sib hundreds of kilometers away from the *I. persulcatus* range (sample Est3974-1) may be a result of transport of ticks by birds or mammals from east to west in Estonia. In Estonia, a role for migratory birds in the dispersal of TBEV infected nymphs from breeding areas in Estonia, Finland and Northwest Russia along the route to the wintering areas in Central and South Africa has been demonstrated [33].

In the present study we also detected another kind of exchange involving TBEV subtype and tick vector: the presence of TBEV-Eur subtype in *I. persulcatus* ticks in an area of co-circulation of the two tick species in Latvia. Similar findings were previously reported from Latvia [24] and recently from Finland, 200 km north of *I. ricinus* range in area where only *I. persulcatus* is distributed [12]. Moreover, identical or closely related strains of TBEV-Eur were isolated from bank voles (*Myodes glareolus*) in the same Finnish area, which indicated the establishment of a new TBEV-Eur focus without its natural tick vector *I. ricinus,* and the historical data allowed the authors to evaluate age of this focus to be 50 years [12]. Similar unusual TBEV-Eur foci have been reported in South Korea, appr. 7000 km away from the European range of TBEV-Eur subtype circulation. TBEV-Eur strains were detected in *Haemophysalis longicornis*, *H. flava* and *Ixodes nipponensis* as well as in wild rodents [34], [35], [36], [37]. How and when strains of the TBEV-Eur subtype were introduced in South Korea remains unclear, but TBEV strains related to the TBEV-Eur subtype were detected by molecular hybridization of nucleic acids in *I. persulcatus*, rodents and humans in the Eastern and Western Siberia as well as in the Ural [30]. Another example of unusual establishment of TBEV-FE foci was reported in Crimea, about 3000 km away from the known TBEV-FE circulation area [5], [38]. It was suggested that moving wild boars (*Sus scrofa*) in 1957 by aeroplane from Far-East of Russia and acclimatization of the animals in Crimea may explain the introduction of TBEV-FE strains into new foci [38], [39]. Such a rapid move of animals together with attached *I. persulcatus* ticks probably infected by TBEV might introduce the virus into the local *I. ricinus* population in which the virus has been maintained until the present date.

Although TBEV TBEV-Sib and TBEV-Eur are more frequently detected in a non- natural tick vector, *I. ricinus* or *I. persulcatus*, respectively, strains of TBEV-Sib and TBEV-Eur still have a restricted range of circulation, and there is no apparent move in a west-east direction and exchange of tick species.

Detection and isolation of TBEV in *Dermacentor* tick species are not rare; in natural foci the virus has been detected in *D. reticulatus* and *D. marginatus* [38], [40]. Recently, a higher prevalence of TBEV in *D. reticulatus* as compared to *I. ricinus* was found in Poland [14] in an area sympatric for both tick species. Larvae of *D. reticulatus* (or other tick species) may be infected by local TBEV strains circulating in the same area during co-feeding with *I. ricinus* or *I. persulcatus* nymphs on the same mammals as demonstrated in Udmurtia [41]. Maintenance of TBEV by *D. reticulatus* in natural foci in the absence of *Ixodes* ticks is very doubtful [42], but this species may support TBEV circulation in *I. ricinus* populations.

To conclude, in this study we reported the TBEV prevalence in questing ticks in the Baltic countries, and we found that strains of TBEV-Sib and TBEV-Eur may be detected, not only in the natural tick vectors, but also in sympatric tick species. While the significance of these findings is unclear at the present, further investigations may clarify if there is a spill-over of TBEV from the natural tick vector to a co-existing tick species in the same area, or the beginning of virus adaptation to a new tick species and as a result of a move to new geographical areas.

## Supporting Information

Table S1 **GenBank accession numbers of TBEV strains used in phylogenetic analysis.**
(DOCX)
