# Supplementary material for: Detection and Characterization of Tick-Borne Encephalitis Virus in Baltic Countries and Eastern Poland
Source: PLoS One. 2013 May 1;8(5):e61374. doi: 10.1371/journal.pone.0061374 (PMC3641128; doi:10.1371/journal.pone.0061374)
Supplement: Table S1 — GenBank accession numbers of TBEV strains used in phylogenetic analysis. (DOCX) [file pone.0061374.s001.docx]

Table S1. Accession numbers of TBEV strains used in phylogenetic analysis

| **Strain** | **Accession no.** | **Geographical origin** |
| --- | --- | --- |
| **The E gene** |  |  |
| LGTV | Y07863 | United Kingdom |
| Lat9793 | AJ319585 | Latvia |
| Kumlinge | GU183380 | Finland |
| Simo-48 | HQ228015 | Finland |
| Simo-38 | HQ228014 | Finland |
| Lat8369 | AJ319584 | Latvia |
| Lat11686 | AJ319582 | Latvia |
| Lit-1-00 | AJ414703 | Lithuania |
| TBE263 | U27491 | Austria |
| KrM215 | EU276111 | South Korea |
| KrM93 | EU276109 | South Korea |
| KOR-07-046 | FJ972625 | South Korea |
| KrM 219DQ | DQ988684 | South Korea |
| KrM 216 | EU276112 | South Korea |
| KrM 219 | EU276113 | South Korea |
| KrM 213 | HM535610 | South Korea |
| Lat8110 | AJ319583 | Latvia |
| Neudoerfl | U27495 | Austria |
| Lat12718 | AJ319586 | Latvia |
| Est3051 | DQ393775 | Estonia |
| HYPR | U39292 | Czech Republic |
| Semeks | AF224665 | Ukraine |
| 2614 | GU060546 | Russia, South-Western Siberia |
| 1284 | EU443278 | Russia, West Siberia |
| IR99-1M4 | AB049349 | Russia, Irkutsk |
| IR99-2F7 | AB049352 | Russia, Irkutsk |
| Kolarovo-2008 | FJ968751 | Russia |
| IR99-1M1 | AB049348 | Russia, Irkutsk |
| Aina | AF091006 | Russia, Irkutsk |
| Vasilchenko | M97369 | Russia, Novosibirsk |
| EK-328 | DQ486861 | Estonia |
| Est54 | DQ393773 | Estonia |
| Lat1-96 | GU183382 | Latvia |
| Vologda-509-75 | FJ214142 | Russia, Vologda |
| Volkhov-2-43 | FJ214148 | Russia, Volkhov |
| Kokkola-9 | DQ451287 | Finland |
| Kokkola-39 | DQ451290 | Finland |
| Oshima | AB001026 | Japan |
| Crimea | AF091008 | Ukraine |
| KH98-10 | AB022297 | Russia, Khabarovsk |
| Sofjin | X07755 | Russia, Primorsky krai |
| LatRK1424 | AF091016 | Latvia |
| N132 | AF091013 | Russia, Vladivostok |
| Tblood | AF091019 | Russia, Perm |
| GGEV | X77732 | Spain |
| SSEV | X77470 | Spain |
| TSEV | DQ235151 | Turkey |
| Est610, Est615, Est620, Est728, Est730, Est732, Est733, Est736, Est876, Est3468, Est3469, Est3625, Est3626, Est3710 | KC660813 | Estonia |
| Est617 | KC660814 | Estonia |
| Est741 | KC660815 | Estonia |
| Est3512-1, Est746 | KC660816 | Estonia |
| Est3974-1 | KC660817 | Estonia |
| Est1045 | KC660818 | Estonia |
| Est758, Est759 | KC660819 | Estonia |
| Est1043, Est1044, Est1048 | KC660820 | Estonia |
| Est3479 | KC660821 | Estonia |
| Est1047 | KC660822 | Estonia |
| Est1530 | KC660812 | Estonia |
| Est1149 | KC660811 | Estonia |
| Est2270 | KC660810 | Estonia |
| Lat103, Lat104, Lat184, Lat185 | KC660801 | Latvia |
| Lat1643 | KC660802 | Latvia |
| Lith129 | KC660803 | Lithuania |
| D49 | KC660804 | Poland |
| D60 | KC660805 | Poland |
| J49 | KC660809 | Poland |
| J103 | KC660806 | Poland |
| Si218 | KC660807 | Poland |
| B273 | KC660823 | Poland |
| B249 | KC660808 | Poland |
| **NS3 gene** |  |  |
| LGTV | Y07863 | United Kingdom |
| TSEV | DQ235151 | Turkey |
| GGEV | DQ235153 | Greece |
| Korppoo-259 | HM051181 | Finland |
| Neudoerfl | U27495 | Austria |
| Isosaari-5 | HM051190 | Finland |
| FinHuman2007 | HM051182 | Finland |
| 263 | DQ153877 | Czech Republic |
| Salem | FJ572210 | Germany |
| KrM93 | HM535611 | South Korea |
| KrM213 | HM535610 | South Korea |
| HYPR | U39292 | Czech Republic |
| FinHuman2008 | HM051183 | Finland |
| SSEV | DQ235152 | Spain |
| Kokkola-84 | HM051186 | Finland |
| EK-328 | DQ486861 | Estonia |
| Karelia-94 | HM051184 | Russia |
| Karelia-108 | HM051185 | Russia |
| Zausaev | AF527415 | Russia |
| Vasilchenko | AF069066 | Russia |
| Kolarovo-2008 | FJ968751 | Russia |
| Primorye-69 | EU816453 | Russia |
| Primorye-253 | EU816451 | Russia |
| Primorye-332 | AY169390 | Russia |
| Primorye-212 | EU816450 | Russia |
| Primorye-18 | GQ228395 | Russia |
| Primorye-90 | FJ997899 | Russia |
| Primorye-86 | EU816455 | Russia |
| Primorye-270 | EU816452 | Russia |
| Sofjin-HO | AB062064 | Russia |
| Primorye-89 | FJ906622 | Russia |
| Primorye-94 | EU816454 | Russia |
| Dalnegorsk | FJ402886 | Russia |
| Glubinnoe/2004 | DQ862460 | Russia |
| Senzhang | JQ650523 | China |
| Kavalerovo | FJ402885 | Russia |
| Est610 | KC660828 | Estonia |
| Est615, Est617, Est620, Est730, Est732, Est733,  Est736, Est741, Est3625, Est3626, Est3469 | KC660827 | Estonia |
| Est1043, Est1044, Est1048, Est1039, Est3479 | KC660829 | Estonia |
| Est758 | KC660830 | Estonia |
| Est759 | KC660831 | Estonia |
| Est1045 | KC660826 | Estonia |
| Est746, Est3512-1 | KC660825 | Estonia |
| Est876 | KC660824 | Estonia |
| Est2270 | KC660833 | Estonia |
| Est1149 | KC660832 | Estonia |
| Est1530 | KC660834 | Estonia |
| Lat103, Lat104 | KC660836 | Latvia |
| Lat184, Lat185 | KC660837 | Latvia |
| Lat1643 | KC660838 | Latvia |
| Lith129 | KC660840 | Lithuania |
| Lith130 | KC660839 | Lithuania |
| D49 | KC660844 | Poland |
| D60 | KC660845 | Poland |
| J49 | KC660835 | Poland |
| J99 | KC660846 | Poland |
| J103 | KC660847 | Poland |
| Si218 | KC660841 | Poland |
| B249 | KC660843 | Poland |
| B273 | KC660842 | Poland |
